# Adolescents' Affective and Physiological Regulation Shape Negative Behavior During Challenging Equine Assisted Learning Activities

**DOI:** 10.3389/fvets.2018.00300

**Published:** 2018-12-04

**Authors:** Patricia Pendry, Alexa M. Carr, Jaymie L. Vandagriff

**Affiliations:** Department of Human Development, Washington State University, Pullman, WA, United States

**Keywords:** equine facilitated learning (EFL), equine assisted learning (EAL), momentary emotion, HPA axis, cortisol, observed behavior, adolescents

## Abstract

This study examined associations between adolescents' (*N* = 59; *M*_age_ = 11.63) diurnal and momentary activity of the Hypothalamic Pituitary Adrenal (HPA) axis as marked by salivary cortisol, and affective and behavioral responses to their first, mounted equine assisted learning (EAL) activity. The introduction to riding occurred during the fifth week of an 11-week EAL program for at-risk and typically developing adolescents. Before the 11-week program began, participants collected 6 salivary cortisol samples at prescribed times (wakeup, 4 p.m., bedtime) over 2 days, from which indices of diurnal cortisol activity were derived. Six weeks later, on the day of their first mounted activity in week five, participants provided three salivary cortisol samples, reflecting their basal cortisol level at the end of their regular school day, and their cortisol levels linked to the beginning and end of their first ride. Participants reported on positive and negative emotion immediately before mounting the horse, and immediately after dismounting, using an 11-item survey. Using a 43-item checklist, three independent observers rated participants' behavior throughout the 90-min session. Regression analyses showed that adolescents with higher cortisol levels immediately before mounting reported higher levels of negative emotion (*B* = 0.350, *p* = 0.041) and lower levels of positive emotion (*B* = −0.697, *p* = 0.013), while basal levels and potential dysregulation of cortisol diurnal patterns were controlled. Greater *cortisol reactivity* in response to 10 min of riding was linked to higher negative (*B* = 2.95, *p* = 0.001), and lower positive emotion (*B* = −3.73, *p* = 0.007) after dismounting. Higher levels of pre-ride negative emotion (*B* = 5.50, *p* = 0.046), and lower levels of post-ride positive emotion (*B* = −5.17, *p* = 0.027), and an increase in cortisol reactivity in response to riding (*B* = 0.242, *p* = 0.049), predicted higher levels of negative behavior during the 90-min session that day. These findings show that participants' HPA axis activity informs their program experience and behavior. Results suggest that EAL facilitators need to employ strategies to down regulate adolescents' physiological and affective arousal during mounted sessions to prevent and redirect negative behavior.

## Introduction

Equine assisted learning (EAL), which combines experiential learning, interaction with equines, and life skills education to increase participants' affective, physiological, and behavioral regulation, has seen a significant increase in use and popularity; in 2016, the Professional Association of Therapeutic Horsemanship International (PATH Intl.) provided Equine Assisted Learning programs at 357 of its 881 member service centers, up from 185 in 2009 (1). Programs that incorporate EAL are appealing because they are well-suited as community or school-based prevention programs, require less training and expertise in comparison to psychotherapy (equine-assisted psychotherapy), and enjoy positive public perception. Also, although limited in number, there is a small but promising number of studies featuring causal designs suggesting participation in EAL has positive effects on adolescents' self-perceived social support (2), adolescent social competence and behavior (3, 4) and adolescents' basal activity of the Hypothalamic Pituitary Adrenal (HPA) axis, as measured by salivary cortisol levels (5).

At first glance, one might assume that increased use of EAL is informed by the prevailing model guiding preventive intervention research, the Preventive Intervention Research Cycle. According to this model, interventions are developed with a comprehensive theoretical and empirical understanding of the target issue, tested for efficacy under tightly controlled research conditions, then examined in real-world settings for effectiveness in broader populations, and finally disseminated for wide-spread implementation. It is a significant concern that this sequencing has not occurred for EAL, which is widely promoted and implemented, despite the fact that the number of causal studies are limited. In fact, little is known about whether, how, under which conditions, and for whom, EAL programs facilitate safe, efficient and effective improvement of affective, physiological, and behavioral regulation.

Based on a survey on EAL and Equine Assisted Therapy (EAT) methodologies Nelson et al. (6) suggested that in order to move EAT and EAL into mainstream professional practice, the theoretical and empirical underpinnings of activities must be defined more clearly. In particular, given that there are no standardized, evidence-based curricula, widely-accepted implementation protocols, or even broad principles guiding EAL implementation, we do not know which EAL activities are essential to achieve desired effects on targeted outcomes in a given population. Furthermore, the most effective ways to enhance learning services through interacting with horses and the equine environment are not yet known to us. In fact, we do not know much about the ways in which one of the most common and popular EAL activities—riding—is best implemented to facilitate an effective experience for populations who vary in age, risk-status, regulatory ability, and prior horse exposure. Specifically, given that EAL programs often target at-risk populations, it would be helpful to understand the role of participants' affective and physiological regulatory abilities commonly associated with risk status, in shaping their moment-to-moment experiences and responses to EAL activities, especially activities that challenge those abilities. Understanding the dynamic interplay between participants' regulatory characteristics and responses in the context of a mounted activity can help equine facilitators anticipate, recognize, respond and redirect signs of participants' arousal, cognitions, emotion, and behavior to enhance participants' subjective perceptions about EAL experiences, which may enhance treatment effects.

Among numerous indicators of physiological regulation, an examination of diurnal, basal, and momentary response patterns of the Hypothalamic Pituitary Adrenal (HPA) axis holds particular relevance for the field of EAL. Marked by cortisol, which can be measured conveniently and unobtrusively in naturalistic settings using salivary sampling, the HPA axis is one of the body's most relevant stress-sensitive systems on the basis of its connection to social, emotional, and psychological events (7). In the EAL context, events that may activate HPA axis activity include exposure to environmental stressors (e.g., riding a 1,200-pound animal, performing a new task with an audience) and social support systems (e.g., encouragement from staff and peers, horse responding to cues).

Typically, healthy individuals show a pronounced diurnal pattern of cortisol, in which levels are highest in the morning soon after waking, drop rapidly in the first few hours after waking, then continue to drop more slowly, reaching a low point around midnight (8, 9). In fact, time of day has been shown to account for ~70% of the variation in cortisol levels (10). There is a substantial body of research on associations between diurnal cortisol activity and individuals' trait-like affective and behavioral characteristics. For example, in adolescents flatter cortisol slopes from wakeup to bedtime have been observed for males with high levels of neuroticism (11) and in youth who have high trait loneliness (12). Moreover, in a prospective longitudinal study, adolescents with a higher baseline Cortisol Awakening Response (CAR) are significantly more likely to experience an episode of major depression over the following year (13); yet, those who experience current or past major depressive episodes exhibit flatter cortisol curves. Depression itself is thought to further alter the functioning of the HPA axis (14), further capturing the dynamic interaction between affective experiences and HPA functioning over time.

Beyond diurnal patterns of cortisol production attributable to the time of day, the remaining variation in cortisol levels is conceptualized as cortisol reactivity, which is determined by responding to momentary influences and events, including social or psychological stressors and supports. Examining momentary reactivity in the context of EAL is reasonable, as there is evidence to suggest that momentary changes in adolescent cortisol levels are associated with momentary, within-person changes in emotion states and social environments. For example, Adam (13, 14) found that momentary cortisol levels were higher than expected for that time of day at moments when individuals were experiencing negative emotion (e.g., anger, worry, stress), and lower when they were experiencing positive social emotions. This has direct relevance for EAL context, as it is likely that EAL activities may evoke negative and positive emotions in participants depending on the activity, the participant's characteristics, as well as the context in which the as activity occurs.

Furthermore, there is evidence to suggest that the presence of social support from a trusted adult may buffer elevations in momentary cortisol during times of fear or emotional distress (15). Although adolescence marks a time when caregivers' capacity to buffer their children's HPA response to stressors appears to decline (16), due to increases in cognitive complexity adolescents are increasingly able to represent others, such as non-family adults and peers, as sources of support (17), providing opportunity for those involved in EAL, including facilitators, volunteers, and fellow participants, to play a role in the development of coping abilities in this context.

Whether adolescents' momentary mood states alter their levels of cortisol, or whether their hormonal states influence their momentary mood states remains undetermined. For example, there is a large body of experimental evidence showing that exposure to stressful situations increases cortisol levels (18, 19), as well evidence demonstrating that experimentally manipulated changes in cortisol are associated with changes in affective and cognitive state (20). It is thus likely that adolescent mood states and cortisol levels transact dynamically and continuously during EAL activities, particularly when experiencing a novel, potentially stressful event, such as engaging in a first mounted activity.

While individual differences in the size and duration of cortisol reactivity are thought to be relevant for affective and behavioral regulation, it is incorrect to assume that downregulation of cortisol activity and reactivity should always be an inherent goal of EAL, as this is dependent on the population under study and the targeted outcome. Researchers studying associations between HPA axis activity and emotional pathology in adolescence have theorized that adolescents with elevated basal cortisol levels and/or a tendency toward greater HPA reactivity to social and emotional challenges may be at greater risk for the development of internalizing problems, including depression and anxiety disorders (21, 22). As such, attempts to downregulate basal cortisol and lower cortisol reactivity may be warranted for some adolescents reporting symptoms of depression and anxiety to prevent the development of clinical levels of either disorder. On the other hand, there is evidence to suggest that physiological regulation may be different for individuals with developmental disorders or psychopathology. For example, individuals with ADHD tend to have low basal levels, as well as low momentary arousal in response to activities, and may as such benefit from program activities that aim to increase physiological arousal (23), a feature that would be counterproductive when working with adolescents with autism, who tend to have over arousal of cortisol levels (24). In sum, given the limited knowledge about the efficacy of EAL to affect momentary down- or upregulation of HPA axis activity, it is somewhat premature to advise practitioners about in- or exclusion of up- or downregulation activities purely based on the etiology of the population served. In fact, examining associations between participants' moment-to-moment emotion, diurnal, basal, and moment-to-moment reactivity of HPA-axis activity in the context of a common, yet challenging EAL activity, and the contributions of these responses to participants' behavioral regulation during the activity is an important first step toward increasing our knowledge. Although there is theoretical rationale to expect individual differences in physiological and affective responding to the stressors and support inherent in EAL settings, there is currently no prior study that has examined these processes on the momentary, experiential level.

The first aim of this study was to examine moment-to-moment emotion states of 10–14-year-old adolescents before (i.e., anticipatory emotion) and after (i.e., post-ride emotion) their first ever mounted EAL activity. We selected the first mounted activity for closer examination as it was expected to evoke different reactions with regards to participants' perceptions of stress and support, which we hypothesized would result into varying levels of HPA activation, differences in moment-to-moment emotion and observed behavior. This first mounted activity took place during the fifth week of an 11-week EAL program which reduces the likelihood that affective and physiological arousal observed in our study occurred merely in response to the novelty of being exposed to equines. The second aim was to model these emotion states by examining contributions of adolescent characteristics (e.g., gender, age, referral status) and indices of physiological regulation (e.g., basal cortisol levels, dysregulation of diurnal patterns, and momentary cortisol reactivity in response to riding). The third aim focused on the extent to which participants' emotion and arousal in anticipation and response to riding informed the quality of observed adolescent behavior during their first mounted activity.

## Material and Methods

All study procedures involving human participants were conducted in accordance with the ethical standards of the institutional research committee and with the 1964 Helsinki declaration and its later amendments or comparable ethical standards. All procedures performed involving animals were in accordance with the ethical standards of the University's Institutional Animal Care and Use Committee.

### The EAL Program and Focus Session

The EAL session under study was conducted during the fifth week of an 11-week EAL program aimed at enhancing adolescents' social competence and reducing stress. The overall program consisted of eleven weekly, 90-min sessions of individual, team, and group-focused equine assisted activities, which were conducted at a PATH Intl. Premier Accredited Center in a university setting. The curriculum utilized in the study was designed by several individuals including a PATH Intl. certified instructor, a licensed counseling psychologist, and a developmental psychologist. Each of these individuals had participated in a range of equine assisted learning and therapy workshops and trainings. While activities varied from week to week and were based on principles of equitation science (25) and natural horsemanship, the curriculum was not situated in one particular field or perspective. Various underlying principles from the aforementioned models were combined with those based on the developmental stress system literature, learning theories, and counseling perspectives and subsequently incorporated into the activities over the 11-week period. The resulting curriculum featured a combination of mounted and un-mounted activities and horse-human interactions, including observation of equine behavior, engagement in equine management (e.g., grooming), in-hand horsemanship, some riding, and personal and group reflection. The program was implemented by a team of PATH Intl. certified instructors and certified equine specialists, undergraduate students in child-development, education, and animal science, professional counseling psychologists, and graduate-level counseling students. Each weekly session featured eight participants, which were paired up with a peer and engaged in a team that included one equine, an equine specialist, and a facilitator, which remained the same throughout the 11 sessions. Sessions were conducted on weekday afternoons as an after school program and included transportation of participants from school, immediately following their regular school day, to the program site and back using program vans. A full description of the weekly program objectives and activities is provided in Table 1. A full description of the activities conducted during the focus session, as well as the timing of data collection is described below and summarized in Table 2.

**Table 1:** Outline of lesson objectives by week.

**Week**	**Lesson objective**	**Sample activity**
1	Basic safety: Meet horses and staff	Observing horse behavior and herd dynamics
2	Respect: Self, others and horses	Moving horses using 4 phases of direct or indirect pressure^*a*^
3	Communication: Verbal and non-verbal	Leading horses, interpreting horse body language
4	Leadership: Assertive and aggressive cues	Driving^*b*^ activity using body language and phases
5	Trust: Coping with perceptions of stress	Riding and leading
6	Boundaries	Driving activity using indirect pressure
7	Overcoming challenges and building confidence	Desensitizing^*c*^ horses
8	Enhancing self-regulation and relaxation	Horse massage^*d*^, riding
9	Prepare for parents/visitors day	Incorporating horsemanship skills for team challenge
10	Parents/visitors day	Participants “teach” parents horsemanship skills
11	Program wrap up	Obstacle course, riding, and reflection

a *Pressure refers to signaling toward the horse (implied/indirect), pushing with fingers/hand or leg until the desired behavior or the equine is fulfilled (i.e., moving away from the pressure)*.

b *Driving refers to signaling the horse to move forward by pointing toward the desired space, and applying pressure behind the equine's shoulder to “drive” them forward and ahead of the equestrian*.

c *Desensitizing refers to exercises to condition the equine to ignore a stimulus or object, thus avoiding “spooking” of certain gestures or objects*.

d *Horse Massage refers to an exercise taught to participants in which they stroke the horse with slow, rhythmic strokes down the equines' body to “relax” him/her before riding*.

**Table 2:** Approximate timing of activities and associated variables during mounted activity session.

**Approximate time since pickup**	**Activity**	**Data collected/Variable derived**
0:00	Pick-up from school	**Cortisol sample 1 collected** (Reflects cortisol level 25 min prior the end of regular school day—*basal cortisol*)
0:25	Arrival at Barn/Participants assist with retrieving horse/arena set up	
0:30	Participants groom their horse for 10 min and assist with saddling	
0:45	A lesson in correct rein use is given	
~0:50	Participants start mounting one by one under supervision^a^. Once mounted, lead walkers lead horse at walk providing basic instruction on sitting, leg pressure and position until all participants are mounted.	**Pre-ride ESM 1 completed immediately before mounting** (Pre-ride positive and pre-ride negative emotion)
~1.00	Once all participants are mounted they begin riding as a group^b^ performing sitting and stretching exercises. After receiving reins, participants work on halting, walking-on, and directing the horse through various obstacles^c^.	
~1:15	Participants start dismounting one by one under supervision in the order they mounted after 20 min of riding.	**Post-ride ESM 2 completed** (Reflects positive and negative emotion after 20 min of riding) **Cortisol sample 2 collected** (Reflects cortisol level immediately before mounting—*anticipatory cortisol*)
~1:25	Participants help put horses and equipment away	**Cortisol sample 3 collected** (Reflects cortisol level after 10 min of riding—*ride cortisol*)
~1:50	Participants end the session with a group discussion about their experience.	

a *Since each participant received one on one assistance of the lead instructor, outlined start, and end times are approximate. While variation in clocked times of riding, cortisol sampling, and survey completion occurred, timing between activities/events, and cortisol sampling (i.e., 25 min), time between cortisol sample 2 and 3 (i.e., 10 min) and total ride time (i.e., 20 min) was identical for each participants*.

b *Participants had a lead walker at all times throughout their riding experience*.

c *Obstacles included weaving through cones, zigzagging through poles, halting between poles, etc*.

### Recruitment

This study employs data collected from participants recruited in the first year of a 2-year randomized controlled trial on the effects of the 11-week program, in which this study is embedded. Although the EAL program studied was suitable to be administered universally or selectively, the aim of researchers and program facilitators was to recruit selectively, with the aim of recruiting approximately equal numbers of boys and girls per grade, as well as giving priority to adolescents with lower social competence. Program participants were recruited and assessed through distribution of flyers and advertisements in two school districts serving ten schools in two small university communities in the states of Washington and Idaho. Recruitment of participants was also accomplished through advertisements in local newspapers and through soliciting referrals by school counselors and local mental health providers. Adolescents referred by professional counselors were either receiving school counseling services for academic and/or behavioral adjustment issues or had parents who had sought consultation by counseling staff about concerns over their adolescent's exposure to school and/or home-based stress. Criteria for program participation were that (1) parents and adolescents were proficient English speakers, (2) the adolescent did not have physical or developmental disabilities, and (3) attended the 5th through 8th grade.

### Screening and Selection

For purposes of screening and selection, parents first completed a standardized measure of adolescent social competence and reported on exposure to school and/or family-based stress for which they were paid five dollars. Social competence was measured with the DESSA (26), a 72-item measure, originally designed for use in schools, asking parents to indicate how often various adolescent behaviors occurred based on a 5-point Likert scale ranging from 0 (*never*), 1 (*rarely*), 2 (*occasionally*), 3 (*frequently*), to 4 (*very frequently*) over the last month. The DESSA has excellent internal reliability (α_1_ = 0.98) and shows significant, moderate-to-high correlations with two widely-used measures with good psychometric properties, the BERS−2nd Edition (27) and the BASC−2nd Edition (28). The DESSA is a strength-based assessment featuring a social competence composite score by summing scores across 8 subscales including Optimistic Thinking (α = 0.87), Self-Management (α = 0.86), Goal-Directed Behavior (α = 0.89), Self-Awareness (α = 0.82), Social-Awareness (α = 0.86), Personal Responsibility (α = 0.87), Decision Making (α = 0.91), and Relationship Skills (α = 0.93). Screened participants were rank-ordered based on these scores to give priority to adolescents with lower social competence.

Next, selected study participants were randomly assigned to a treatment group starting program participation a week later, or to a waitlisted control group, who started participation 16 weeks later. In year 1 of the trial, 64 participants (*N*_boys_ = 30; *N*_girls_ = 34; *M*_age_ = 10.93 years) were selected for study participation and randomly assigned to an experimental group (*N* = 33) or waitlisted control condition (*N* = 31). Of those, 59 participants attended the session of focus in this study (*N*_boys_ = 27; *N*_girls_ = 32; *N*_referred_ = 10; *M*_age_ = 11.63 years), predominantly White (81.6%) and of non-Latino or Hispanic ethnicity (88.8%), with the remaining adolescents reporting across racial categories that included more than one race (8%), Asian (3.2%), and American Indian or Alaska Native (1.6%), or unknown race (5.6%). Although this study is embedded in a larger, 2-year causal trial, the analyses featured in this manuscript are not designed to make causal inferences.

### Diurnal Activity of HPA Axis: in-Home Salivary Sampling

Parents and adolescents were instructed and consented/assented in person by the PI. Two weeks before the beginning of the EAL program, they received written and verbal instructions and a hands-on demonstration on how to collect and store salivary samples. In the week before the EAL program started, parents assisted adolescents with the in-home sampling of six salivary cortisol samples using the passive drool method by spitting through a straw into a sterile 1.8 ml cryovial. Saliva was collected three times a day, on each of two consecutive weekdays at prescribed events (immediately upon waking, immediately before bedtime) and at prescribed times (4:00 p.m.) in their own home and as they went about their normal daily lives. Participants and parents recorded the exact time each sample was taken, and completed an activity and event report on each sampling day on their use of steroid-based medication, timing of intake of food and beverages, sleep and wake-times, as well as any unusual circumstances that may have influenced reliable sampling or interpretation (e.g., sickness, exposure to unusual or stressful event) during days of salivary sampling. Substantial efforts (i.e., written, verbal and in-person instruction, hands-on demonstration, and telephone and email reminders) were made to impress upon participants the importance of compliance with the study's sampling procedures, particularly with regards to the timing of saliva sampling and reporting of sampling times, especially those collected upon waking. Compliance was high as 92% of adolescents provided all six diurnal samples as requested. A set of completed samples were retrieved from participants at the beginning of the EAL program session when samples were stored on ice in a cooler for transfer to our laboratory-based freezer for storage at −80 degrees Celsius that day, after visual inspection for blood contamination and sample cataloging.

### Momentary Cortisol Sampling

In addition to calculating diurnal indices of HPA axis activity a week before the start of the program, participants were asked to provide three momentary samples of salivary cortisol during the fifth session, which constitutes the focus of this study. Adolescents provided their first saliva sample under supervision from research assistants, immediately upon being picked-up from school before being transported to the barn. Since it takes ~25 min after an event or stressor for cortisol levels to reach their peak in saliva (29), participants' cortisol levels collected at the time of pickup reflected their HPA axis activity ~25 min *prior* to the end of their regular school day, therefore referred to as *basal cortisol*.

Although the focus session was implemented according to the activity and sampling outline presented in Table 2, each participant's *specific* mounting, riding, dismounting, sampling, and survey activity was individually managed, systematically-timed with a stopwatch, and documented to ensure that the observed associations between riding-related events, levels of cortisol present in saliva, and participants' momentary emotions reflecting riding-related events are empirically justified. Since participants mounted and dismounted one by one, while under direct supervision of the certified lead PATH instructor, each individuals' precise mounting time was used as the anchoring time for determining the salivary sampling time −25 and 35 min later—of the individual's subsequent two cortisol samples. We conceptualized the cortisol parameter collected 25 min after mounting as *anticipatory* cortisol to best capture the dynamic, continuous, and transactional nature of HPA axis activity likely to operate in the context of the mounting process, which constitutes a novel, potentially arousing stressor usually lasting several minutes. The third saliva sample was collected exactly 35 min after mounting (e.g., ride cortisol), which was used to calculate the participants' *cortisol reactivity* by calculating the difference between the third and second sample.

While we recognize that the precise time elapsed between events and peak level of cortisol obtained in saliva is an approximation, it is less important to focus on the absolute values of these variables than it is to consider their value in capturing *change* in participants' cortisol levels in the context of ~10 min of riding, which is informed by participants' perception of the availability of coping resources and support (21). Any factor which influences the individual's perception of themselves and their environment, including their past experience (e.g., risk-status), their emotional and physical traits and states (e.g., moment-to-moment emotion, basal cortisol, diurnal dysregulation), and the nature of their support system (e.g., ability of equine-assisted facilitators to facilitate down regulation of arousal, presence of supportive peers, a responsive, gentle horse) may influence this perception and the associated reactivity. Each participant rode for a total of 20 min and dismounted in the order they mounted. All saliva samples were collected under the supervision of trained research assistants who immediately marked each sample with the exact time and date of collection. Completed samples were stored on ice in a cooler for transfer to our laboratory-based freezer for storage at −80 degrees Celsius that day, after visual inspection for blood contamination and sample cataloging.

### Momentary Emotion Sampling

Measurement of participants' moment-to-moment emotion was based on procedures and measures of the experience sampling method (ESM) (30), which is a method that provides detailed information about participants' subjective interpretations of their experiences in naturalistic settings. Designed to capture individuals' subjective evaluations of events at a particular moment is particularly valuable for studying emotions such as stress, since it is possible to determine a person's stress level at a given moment, as well as to identify specific instances when stress increases or decreases in response to specific events (31). Research examining the quality of ESM data has concluded that these data are reliable and valid when compared with data obtained from other instruments (32, 33). Findings also indicate that respondents are generally truthful in reporting their immediate subjective experiences (34), thus confirming the validity of ESM. Using a protocol from a prior study linking emotional functioning to cortisol levels (35, 36), ESM reports were taken twice during the session under study, once immediately before mounting the horse, and once immediately after dismounting, 20 min later. The 1-min 11-item survey asked participants to endorse on a four-point scale, ranging from 0 (*not at all*), 1 (*a little*), 2 (*somewhat*), to 3 (*very much*), the extent to which they “*felt embarrassed, nervous, overwhelmed, frustrated, stressed, confident, relaxed, excited, happy, proud, and relieved.”* To reduce the potential of participants providing socially desirable answers, each participant was provided privacy while they completed their survey. To reduce the possibility of type 1 error, principal component analyses (with a varimax rotation) were performed revealing two main emotion factors, including negative emotion (i.e., embarrassed, nervous, overwhelmed, frustrated, stressed, and confident and relaxed, which were both reverse coded) and positive emotion (i.e., excited, happy, proud, and relieved). Coefficient alphas for negative and positive emotion were 0.73 and 0.68, respectively. These scores were used to derive at variables capturing positive and negative emotion before riding (i.e., *pre-ride positive emotion, pre-ride negative emotion*) and after riding (i.e., *post-ride positive emotion, post-ride negative emotion*).

### Participant Behavior

For study purposes related to the 2-year RCT in which this study was embedded, participants' positive and negative behaviors were rated weekly after each session using the Animal Assisted Therapy—Psychosocial Session Form (AAT-PSF) (37). Since these ratings had been completed each week, we used rating of behavior obtained at the beginning of the program (Week 1) as a control variable in models predicting behavior observed during the focus week under study. Each adolescent's behavior was independently rated by two program facilitators who worked with the adolescent during the focus session, and a third rater, a research assistant not engaged in the facilitation of human-horse interaction. Raters indicated the extent to which adolescents engaged in 25 positive behaviors (e.g., following direction, accepting feedback, sharing, making eye contact, appropriately assertive) and 18 negative behaviors (e.g., argumentative, fidgeting, withdrawn, hyperactive, resistant) on a six—point Likert scale containing 0 (*none*), 1 (*very low*), 2 (*low*), 3 (*medium*), 4 (*high*), and 5 (*very high*). Summed scores for each participant's positive and negative behaviors were averaged across observers, whose ratings were positively associated as evidenced by a significant intra-class correlation, *r* = 0.829, *p* < 0.001 resulting in a score of *positive and negative behavior* for each participant.

### Data Reduction of Cortisol Values and Parameters Calculation

All samples were analyzed by a professional laboratory specializing in salivary cortisol assaying by enzyme immunoassay. The test used for these assays had a range of sensitivity from 0.007 to 1.8 μg/dl, and average intra- and inter-assay coefficients of variation < 3 and 7%, respectively. Before calculating diurnal parameters, we replaced missing cortisol values with the value of the participant's own cortisol value taken at the same time on the other sampling day, rather than replacing the value with the sample mean. To limit the influence of extremely high or low individual cortisol values, diurnal cortisol values for each time point were winsorized to three standard deviations above and below the mean. The slope value of the adolescent's diurnal cortisol curve was calculated by regressing all six of the participant's cortisol values on his or her sampling time over the 2-day sampling period and the unstandardized coefficients derived from these regression analyses were used as a dependent variable for each participant, effectively controlling for the time of day samples were taken, and for the total time the adolescent was awake. Slope values were utilized in the classification of whether adolescents displayed *cortisol dysregulation*, as indicated by a positive slope, for which an indicator was assigned. As is common when conducting these types of analyses, a natural logarithmic transformation for each cortisol parameter at each time point (e.g., basal cortisol, anticipatory cortisol, and cortisol reactivity) was used to reduce positive skewness typical of this biomarker, followed by standardizing for regression analyses.

## Results

### Comparing Momentary Emotion Before and After Riding

Descriptive characteristics of participants' emotion factors and items are reported in Table 3. Using paired-samples *t*-tests, we compared reports of momentary emotion for each item and factor reported immediately before riding during mounting (i.e., *anticipatory emotion*) to emotion reported immediately following riding for 20 min (i.e., *post-ride emotion*). Given the within-subject nature of these analyses, we calculated a Cohen's *d* effect size for each item and factor while controlling for the correlation between measurements of each item (38).

**Table 3:** Descriptives of momentary emotion before and after 20 min of riding (*N* = 59).

	**Pre-Ride *M* (SD)**	**Post-Ride *M* (SD)**	**ANOVA *p*-value**	**Cohen's *d_*z*_***
**Negative Emotion Factor**^a^	0.578 (0.52)	0.446 (0.56)	0.052	−0.26
Embarrassed	0.193 (0.48)	0.298 (0.71)	0.277	0.15
Confident	2.21 (0.97)	2.48 (0.88)	0.041^*^	0.27
Relaxed	2.25 (0.96)	2.41 (0.93)	0.296	0.13
Nervous	0.948 (1.02)	0.448 (0.88)	0.001^**^	−0.47
Overwhelmed	0.610 (0.98)	0.559 (0.99)	0.695	−0.05
Frustrated	0.170 (0.56)	0.203 (0.61)	0.687	0.05
Stressed	0.586 (0.89)	0.500 (0.92)	0.301	−0.13
**Positive Emotion Factor**	2.04 (0.79)	2.36 (0.82)	0.033^*^	0.28
Excited	2.51 (0.79)	2.36 (0.82)	0.231	−0.16
Happy	2.54 (0.73)	2.56 (0.84)	0.849	0.02
Proud	1.79 (1.10)	2.35 (0.88)	< 0.001^***^	0.55
Relieved	1.19 (1.18)	1.64 (1.26)	0.001^**^	0.44

* *p < 0.05*.

** *p < 0.01*.

*** *p < 0.001*.

a *Confident and relaxed are represented as actual scores, yet were reverse coded to enable factor calculations*.

The overall factor for negative emotion revealed that adolescents' levels of negative emotion were significantly higher immediately before riding compared to their levels of negative emotion after having ridden for 20 min *t*_(58)_ = 1.98, *p* = 0.052, *d*_z_ = 0.26. This was mostly driven by adolescents feeling less nervous *t*_(57)_ = −3.58, *p* = 0.001, *d*_z_ = −0.47 and more confident *t*_(57)_ = 2.09, *p* = 0.041, *d*_z_ = 0.27 after having completed their ride. Results also showed that participants reported significantly higher positive emotion after riding, *t*_(57)_ = 2.19, *p* = 0.033. This was mostly driven by adolescent's reports of feeling proud *t*_(56)_ = 4.16, *p* < 0.001. We also examined individual differences in positive and negative emotion factors by gender and referral status before and after riding. Results suggest that there were no significant differences by gender for positive emotion before, *F*_(1, 57)_ = 0.001, *p* = 0.972, or after riding *F*_(1, 57)_ = 0.770, *p* = 0.384. Similarly, there were no significant differences by gender for negative emotion before, *F*_(1, 57)_ = 0.421, *p* = 0.519, or after riding, *F*_(1, 57)_ = 0.002, *p* = 0.967. With regards to referral status, we noted that the most pronounced difference was experienced in the levels of negative emotion after riding, *F*_(1, 57)_ = 11.78, *p* = 0.001 showing that adolescents who came to the program referred by school counselors reported significantly higher levels of negative emotion after riding, *M* = 0.691, *SD* = 0.87, than those who were non-referred, *M* = 0.341, *SD* = 0.42. As such, in models examining the role of participants' risk-related characteristics on momentary emotion before and after riding, we included referral status.

### Diurnal Cortisol Collected at Pre-Test, Before Start of 11 Week Program

Descriptive analyses on observed cortisol levels and sampling times are described in Table 4. Results show that diurnal cortisol levels for participants collected at pre-test (1 week before the start of the program, 6 weeks before the session under study took place) were considered in the normal range across both sampling days for wakeup cortisol levels *M*_Day1_ = 0.330, *SD* = 0.20, *M*_Day2_ = 0.332, *SD* = 0.22, afternoon cortisol levels *M*_Day1_ = 0.096, *SD* = 0.16, *M*_Day2_ = 0.133, *SD* = 0.31, and bedtime cortisol levels, *M*_Day1_ = 0.107, *SD* = 0.21, *M*_Day2_ = 0.061, *SD* = 0.15. On average, adolescents were awake for a total of 13.57 h per day. The slope value of the adolescent's diurnal cortisol curve, calculated by regressing each individual's cortisol level on his or her sampling time over the 2-day sampling period, was also in the normal range, *M* = −0.019, *SD* = 0.01. Indicators of slope dysregulation were assigned to participants with positive slopes. We did not find any significant differences in the slope of adolescents' diurnal cortisol by gender, *F*_(1, 52)_ = 1.34, *p* = 0.25, or referral status, *F*_(1, 51)_ = 1.21, *p* = 0.28.

**Table 4:** Descriptives of untransformed adolescent cortisol levels (μ/dl).

**Diurnal pretest cortisol**	***M* (SD)**
Cortisol slope	−0.019 (0.01)
Wakeup cortisol _Day1_	0.330 (0.20)
Wakeup cortisol _Day2_	0.332 (0.22)
Afternoon cortisol _Day1_	0.096 (0.16)
Afternoon cortisol _Day2_	0.133 (0.31)
Bedtime cortisol _Day1_	0.107 (0.21)
Bedtime cortisol _Day2_	0.061 (0.15)
Average wakeup time	7.28 (0.78)
Average afternoon time	16.25 (0.78)
Average bedtime time	20.85 (0.75)
**Momentary Riding Week**	***M*** **(SD)**
Sample 1: Baseline cortisol	0.083 (0.061)
Sample 2: Anticipatory cortisol	0.063 (0.052)
Sample 3: Ride cortisol	0.067 (0.099)

### Momentary Cortisol Levels Collected During the Riding Session

Basal cortisol levels reflecting HPA axis activity 25 prior to ending their regular school day (*M* = 0.083, *SD* = 0.061), during mounting (*M*_anticipatorycortisol_ = 0.063, *SD* = 0.052), and after 10 min of riding (*M*_ridecortisol_ = 0.067, *SD* = 0.099) are also reported in Table 4. The reported mean levels and standard deviations reflect untransformed cortisol values, which were transformed to normalize the distribution using a natural logarithmic transformation before further analyses were conducted. There were no statistically significant differences in cortisol levels by gender [*F*_(1, 57)_ = 1.93, *p* = 0.17; *F*_(1, 51)_ = 3.55, *p* = 0.07], referral status [*F*_(1, 57)_ = 1.77, *p* = 0.19; *F*_(1, 51)_ = 0.238, *p* = 0.58], or horse experience prior to starting the program [*F*_(1, 57)_ = 3.02, *p* = 0.09; *F*_(1, 51)_ = 0.313, *p* = 0.63], respectively.

### Physiological Contributions to Positive and Negative Momentary Emotion Before Riding

Using hierarchical linear regression, we first examined associations between participants' basal cortisol levels, anticipatory cortisol, cortisol dysregulation, referral status, and positive and negative emotion reported immediately before riding during mounting (Table 5). In predicting negative momentary emotion before riding (pre-ride negative emotion), results show that higher levels of anticipatory cortisol were significantly associated with higher levels of negative emotion before riding, β = 0.382, *p* = 0.041. As cortisol variables were logarithmically transformed, these variables will be discussed in terms of percentage change, rather than log-unit change for clarity in interpretation. These results suggest that a 1 SD increase in momentary anticipatory cortisol predicts a statistically significant 46.5% increase in feelings of negative emotion during mounting after basal cortisol levels, cortisol dysregulation, and referral status were accounted for.

**Table 5:** Regression analyses predicting momentary emotion during riding session^a^.

	**Unstandardized B**	**SE**	**β**	***p*-value**	***Interpretation^*b*^***
**Pre-Ride Negative Emotion (*****R***^2^ = **0.265)**					
(Constant)	0.822	0.775		0.294	
Anticipatory Cortisol	0.350	0.167	0.382	0.041^*^	46.52% increase in negative emotion
Basal cortisol _pickup_	−0.127	0.176	−0.133	0.472	
Cortisol dysregulation	0.432	0.294	0.183	0.148	
Referred	0.153	0.172	0.111	0.377	
**Pre-Ride Positive Emotion (*****R***^2^ = **0.145)**					
(Constant)	2.03	1.25		0.112	
Anticipatory Cortisol	−0.697	0.270	−0.508	0.013^**^	39.83% decrease in positive emotion
Basal cortisol _pickup_	0.618	0.285	0.431	0.034^**^	53.88% increase in positive emotion
Cortisol dysregulation	0.047	0.475	0.013	0.922	
Referred	−0.187	0.278	−0.090	0.505	
**Post-Ride Negative Emotion (*****R***^2^ = **0.398)**					
(Constant)	0.664	0.784		0.401	
Cortisol Reactivity _Riding_	2.95	0.856	0.395	0.001^**^	48.44% increase in negative emotion
Basal cortisol _pickup_	−0.057	0.122	−0.054	0.643	
Cortisol dysregulation	−0.084	0.284	−0.033	0.767
Referred	0.507	0.169	0.338	0.004^*^	
**Post-ride positive emotion (*****R***^2^ = **0.193)**					
(Constant)	3.35	1.21		0.008	
Cortisol reactivity _Riding_	−3.73	1.31	−0.375	0.007^*^	31.27% decrease in positive emotion
Basal cortisol _pickup_	0.103	0.187	0.075	0.583	
Cortisol dysregulation	0.029	0.437	0.008	0.948	
Referred	−0.097	0.261	−0.048	0.713	

* *p < 0.05*.

** *p < 0.01*.

^***^p < 0.001.

a *Final presented models control for participant age, gender, and whether the participant was a horse novice*.

b *Due to the logarithmically transformed independent variable (i.e., natural log of cortisol values), the inverse function of that transformation (i.e., exponential function) was applied prior to calculating the effect size (i.e., percent change) to return each coefficient to its value*.

Modeling of positive momentary emotion before riding demonstrated that higher levels of anticipatory cortisol significantly predicted lower feelings of positive emotion before riding, β = −0.508, *p* = 0.013. These results suggest that a 1 SD increase in anticipatory cortisol predicts a statistically significant 39.8% decrease in feelings of positive emotion. Interestingly, basal cortisol at pickup was positively associated with significantly higher levels of positive emotion before riding, β = 0.431, *p* = 0.034 (*d* = 53.9%) suggesting that higher basal cortisol levels that afternoon may have served to help the body respond to the stressor in preparation for the event in ways that inform participants' ability to mobilize biological resources (e.g., metabolic functioning, blood glucose levels, blood pressure) even though the size of the acute response as they were mounting their horse may have informed feelings of overwhelm, which may have reduced positive emotions of enjoyment and confidence. Interestingly, neither cortisol dysregulation nor referral status statistically predicted pre-ride levels of positive emotion.

### Effects of Cortisol Reactivity on Positive and Negative Momentary Emotion After Riding

Next, we examined the extent to which participants' *cortisol reactivity* in response to 10 min of riding predicted participants' positive and negative perceptions about their first ride, which were surveyed 10 min later after dismounting. Consistent with literature on contributions of both diurnal, basal, and momentary cortisol levels to affective states, basal cortisol levels and cortisol dysregulation were included in the model, as was referral status. Greater cortisol reactivity in response to riding significantly predicted higher levels of negative emotion after dismounting the horse, β = 0.395, *p* = 0.001 showing that a 1 SD increase in cortisol reactivity predicted a 48.4% increase in feelings of negative emotion after the first riding activity had ended. Additionally, referral to the program significantly predicted higher levels of negative emotion after riding, β = 0.338, *p* = 0.004. Modeling post-ride positive emotion revealed that greater cortisol reactivity in response to the first 10 min of riding predicted a statistically significant decrease in feelings of positive emotion after riding, β = −0.375, *p* = 0.007, suggesting a 31.3% decrease in positive emotions in response to a 1 SD increase in reactivity. Neither basal cortisol, cortisol dysregulation nor referral status statistically predicted feelings of positive emotion after riding. These findings suggest that greater amounts of acute, momentary activation of HPA axis activity in response to a challenging yet perceived joyful activity—riding—heightens negative emotions and dampens positive perceptions about the experience. In addition, while children who were referred and perceived as “at-risk” did not have greater dysregulation of patterns of diurnal cortisol, they were clearly less able to regulate negative affective arousal in the context of these experiences.

### Predicting Negative Behavior During Riding Session

In addition to better understanding the contributions of physiological arousal to affective responses to a common EAL activity, we were interested in examining the extent to which participants' subjective experiences informed their negative behavior during the session. We controlled for gender in the model as descriptive analyses determined that boys were reported to have significantly lower levels of positive behavior *M*_boys_ = 75.85, *SD* = 16.13, compared to girls *M*_girls_ = 86.44, *SD* = 16.25. Additionally, boys displayed significantly higher levels of negative behavior, *M*_boys_ = 14.91, *SD* = 11.88, than girls, *M*_girls_ = 6.92, SD = 5.62).

In our first model (not shown, but discussed in text) we modeled the relationship between positive and negative emotion before and after riding on negative behavior while controlling for gender and age, and found that increased negative emotion measured before mounting significantly predicted higher levels of negative behavior during the riding session, β = 0.361, *t*_(57)_ = 2.35, *p* = 0.023. Additionally, increased feelings of positive emotion after 20 min of riding significantly predicted lower levels of observed negative behavior, β = −0.546, *t*_(57)_ = −2.88, *p* = 0.006. Feelings of positive emotion in anticipation to riding, and feelings of negative emotion in response to riding did not significantly predict observed negative behaviors for this session and were thus excluded in the final model, model 2, discussed below, and presented in Table 6.

**Table 6:** Regression analysis predicting negative behavior during first riding session (*N* = 59).

**(*R*^2^ = 0.387)**	**Unstandardized B**	**SE**	**β**	***p*-value**	**Interpretation*^*a*^***
(Constant)	10.24	5.03		0.047	
Pre-ride negative emotion	5.50	2.69	0.293	0.046^*^	
Pre-ride positive emotion	2.23	1.83	0.178	0.229	
Post-ride negative emotion	−5.68	2.92	−0.328	0.058	
Post-ride positive emotion	−5.17	2.28	−0.398	0.027^*^	
Cortisol reactivity _Riding_	31.30	15.54	0.242	0.049^*^	27.37% increase in negative behavior
Cortisol dysregulation	2.57	5.01	0.058	0.611	
Baseline negative behavior	0.509	0.149	0.392	0.001^***^	

* *p < 0.05*.

^**^p < 0.01.

*** *p < 0.001*.

a *Due to the logarithmically transformed independent variable (i.e., natural log of cortisol values), the inverse function of that transformation (i.e., exponential function) was applied prior to calculating the effect size (i.e., percent change) to return each coefficient to its value*.

The final model (Table 6) integrates additional participant characteristics used in previous analyses while also incorporating relevant indices of HPA axis activity. Increased feelings of negative emotion in anticipation of riding—measured immediately before mounting—significantly predicts negative behavior during the riding session, β = 0.293, *t*_(57)_ = 2.05, *p* = 0.046; and increased feelings of positive emotion after riding significantly predicted lower levels of observed negative behavior, β = −0.546, *t*_(57)_ = −2.88, *p* = 0.006. We also see evidence of HPA-axis reactivity contributing to observed reports of negative behavior. A 1 SD increase in cortisol reactivity in response to 10 min of riding predicted a 27% increase in observed negative behavior during this equine assisted learning program session. Also, higher levels of observed negative behavior during the first program session were associated with a statistically significant increase in levels of observed negative behavior during the first mounted program session. These findings suggest that negative perceptions in anticipation of riding for the first time, which are influenced by basal levels of cortisol on that day, along with increased physiological and affective arousal in response to riding, increased the negative behavior of EAL participants.

## Discussion

The main objective of this study was to examine the associations between adolescents' physiological characteristics, affective experiences, and behaviors during a novel equine activity approximately halfway through the 11-week program. In our examination of the emotional states of adolescents before and after their first mounted equine activity of the program, we found that negative emotion significantly decreased and positive emotion significantly increased. These findings echo findings by Frederick et al. (39), who found that at the end of a 5-week EAL program, academically-at-risk adolescents randomly assigned to additional EAL activities reported higher levels of hope and lower feelings of depression. These findings support the overall notion that getting to ride a horse can be enjoyable, while participants' experiences throughout an EAL session vary depending on the task, as well as the subjective experiences of that adolescent.

Next, we examined the extent to which adolescent characteristics and diurnal and momentary indices of physiological regulation predicted adolescent positive and negative emotion in anticipation and response to riding. Before riding, we found that higher levels of negative emotion were significantly associated with increased momentary cortisol levels, whereas higher levels of positive emotion were significantly associated with lower levels of momentary cortisol before riding and higher levels of afternoon basal cortisol. After the riding activity was completed, higher levels of negative emotion were significantly associated with higher cortisol reactivity in response to 10 min of riding, and being referred to the program, whereas higher levels of positive emotion were significantly associated with lower levels of cortisol reactivity in response to riding.

The presence of individual differences in cortisol reactivity to negative emotion in naturalistic settings is not surprising given the known role of differences in developmental histories, perceptions of stressors, and coping resources for individual differences in cortisol reactivity. Prior research found that the size of the HPA axis response to challenges intended to stimulate corticotropin-releasing hormone had a significant genetic component, whereas individual differences in cortisol reactivity to laboratory-based psychosocial stressors did not (40). Regardless of its origins, variability in cortisol responsivity to negative emotion and events experienced in daily life is potentially of considerable clinical interest. It is possible that “high cortisol responders” to this EAL challenge may experience more negative emotion and less positive behavior. Although participation in EAL activities may be generally enjoyable for adolescents, their experience as indicated by their emotional, physiological, and behavioral response at any point in the activity may vary based on HPA-axis activity, individual characteristics and referral status.

Finally, we examined the extent to which participants' positive and negative emotion in anticipation and response to riding informed the quality of observed adolescent behavior during their first mounted activity. We found that higher levels of observed negative behavior were significantly associated with higher levels of negative emotion before riding, lower levels of positive emotion after riding, and greater cortisol reactivity in response to riding for 10 min. Additionally, higher levels of negative behavior on the first day of the program were significantly associated with higher levels of negative behavior during the first mounted EAL session.

Based on these findings, it is important that program instructors and facilitators take into consideration some of the “invisible” individual characteristics that may be informing a participant's EAL experience, particularly during mounted sessions. Their attention should go beyond simply redirecting participant behavior with the intention of promoting safety, peer relations, activity goals, etc., to also recognize the different regulation abilities of the participants during activities that may be arousing to the adolescent. One avenue of accomplishing this may come from the nature of the programs themselves. EAL may provide a unique platform to assist adolescents in better understanding and cuing into their own physiological and emotional needs. Carlsson et al. (41) reported that clients and staff members engaged in equine assisted social work claimed that the horse's ability to cue into and respond to their emotions was influential in allowing themselves to become aware of those emotions. Notschaele (42) suggests that it is the horse's ability to provide moment-to-moment feedback on human's non-verbal communication that provides the framework for a feedback system that humans do not typically encounter. The underlying idea for this feedback loop is further supported by evidence that horses do in fact respond both physically and behaviorally to human's psychological and physical stress (43, 44). With their knowledge of horse behavior, facilitators can thus provide valuable guidance to participants to assist them with downregulation following an arousing experience, by effectively utilizing the horse's behavior as an external feedback indicator. On the other hand, this approach suggests that all horses cope actively or visually with stressors that can occur in this situation. As such, it may place undue responsibility on the horse as an instrument, when in reality it may be unethical to wait for the horse to communicate discomfort related to the participant. In sum, while the horse may offer up recognizable indicators to facilitators that their client is experiencing stress, it is equally important that each program facilitator be able to independently recognize behaviors of stress in their human clients. Fostering increased awareness of the role of HPA axis activity and arousal during program participation may enable program facilitators to better recognize and respond to arousal in their clients. With the duel knowledge of indicators of stress in both humans and horses, facilitators will be able to better respond to human needs, while simultaneously protecting and promoting the wellbeing of the horses they are working with.

By paying attention to the behaviors and cues of *both* the adolescent and horse, program staff have the opportunity to take a more active role in facilitating the downregulation of aroused adolescents during mounted activities. At times arousing circumstances may require the highlighting of positive actions (i.e., “Nice job rewarding your horse for standing still while mounting”), naming and discussing “negatives” (i.e., “I wonder whether the horse can sense that you are nervous; what do you think?”), or directly instructing the adolescent to use the horse as a measure of their downregulation (i.e., “It seems like you may be gripping your horse with your legs which may be telling your horse that you want to go faster, even though you are also asking your horse to slow down. Let's see if your horse will relax with you; take a deep breath and relax your legs when you exhale”). It is important that facilitators and instructors of EAL programs are aware that participants may not be able to regulate their behavior (i.e., listen to direction, sit still, not pull on the reins) because they are preoccupied and possibly impaired in their own affective and physiological arousal in response to what they may perceive as a psychological or physical threat. As such, rather than focusing on changing the negative behavior *per se*, facilitators may need to focus on helping participants change their arousal and perception as a tool to facilitate desired positive behavior. Doing so will also provide opportunities for the facilitators to point out the horse's desired response, which can be used to reinforce downregulation and positive perception for the participant, functioning as a feedback loop in the physiological, affective, and behavioral domains. Overall, increasing the participants' awareness of emotion, behavior, and cognitions enhances EAL's original goal.

### Strengths and Limitations

Strengths of this study lie in several methodological aspects in the measuring of various dependent and independent variables. First, the collection of the diurnal and momentary cortisol samples and participant moment-to-moment emotion were conducted under a precise, carefully-timed protocol. Although maintaining such a methodological protocol in a naturalistic setting is logistically and procedurally difficult to do, it adds a dynamic set of measures to the expanding literature on EAL programming. In addition, the modeling approach is one of the few in the literature that simultaneously examines diurnal, basal, and momentary activity of the HPA axis. Considering aspects that inform cortisol activity in humans comprehensively thereby informs realistic expectations about participants' ability to control their arousal and reduces the likelihood that participants, especially those with dysregulated patterns, are expected to regulate their behavior more than they are psychologically able. Not recognizing the role of arousal may create the incorrect impression that participants are not willing to follow directions, which can lead to unproductive tension between the participant, their peer, the facilitator, and the horse, particularly when attempts are made to redirect using behavioral approaches (i.e., rewards such as praise; punishment such as sitting out), which tend to be less effective when affective and physiological arousal underlie undesired actions. As such, we believe that these findings provide a much-needed contribution that can inform the practice of EAL for various populations. Another strength of this study is that behavior was assessed by three independent raters, including those unconnected to the EAL implementation and thus unvested in treatment success, the horse or participant. This reduces the likelihood that the ratings were biased by staff exaggerating positive treatment effects.

With regards to study limitations, while the data analyzed in this study were collected under naturalistic settings as a part of a larger randomized controlled trial, the nature of the data does not allow for causal interpretation of our findings. However, these findings may inform future causal experimentation examining “best practices” for program facilitation. Additionally, although we gathered self-reports on emotional states before and after riding, we did not measure participants' appraisal of their experience. Future work examining how participants appraise the situation at hand may better inform the relationship between their physiological reactivity, emotionality, and behavior.

## Conclusion

In summary, this study provides evidence that studying the associations between participants' physiological and affective experiences in response to common EAL activities is informing our understanding of participants' individual differences in behavior. In the long term, gaining a better understanding of how the dynamics of participants' emotions and experiences relate to the dynamics of their cortisol levels and their behavior may help to illuminate the pathways by which EAL can get “under the skin” to influence program success. EAL programming staff have an opportunity to assist adolescents, particularly those at-risk and those experiencing high levels of stress and related symptoms, in downregulating their arousal during mounted program activities to encourage appropriate behaviors, as well as create positive, enjoyable, relaxing experiences. It is important therefore that program staff receive the necessary training and support related to participants' developmental characteristics in addition to their experience with horses in order to not only maintain program safety, but also to promote positive learning opportunities for program participants by highlighting the unique ways in which horses can facilitate these processes.

## Author Contributions

All authors contributed to the manuscript and have read and approved the final version. PP obtained funding, conceived the study concept and design, and drove the analysis and interpretation of data. AC participated in data collection, assisted in the analysis and interpretation of data, and contributed to the manuscript draft. JV critically revised the manuscript.

### Conflict of Interest Statement

The authors declare that the research was conducted in the absence of any commercial or financial relationships that could be construed as a potential conflict of interest.
